# S-nitrosylation regulates mitochondrial quality control via activation of parkin

**DOI:** 10.1038/srep02202

**Published:** 2013-07-16

**Authors:** Kentaro Ozawa, Akira T. Komatsubara, Yuhei Nishimura, Tomoyo Sawada, Hiroto Kawafune, Hiroki Tsumoto, Yuichi Tsuji, Jing Zhao, Yoji Kyotani, Toshio Tanaka, Ryosuke Takahashi, Masanori Yoshizumi

**Affiliations:** 1 Department of Pharmacology, Nara Medical University School of Medicine, Japan; 2 Department of Genomic Drug Discovery Science, Kyoto University Graduate School of Pharmaceutical Sciences, Sakyo-ku, Kyoto, Japan; 3 Department of Molecular and Cellular Pharmacology, Pharmacogenomics and Pharmacoinformatics, Mie University Graduate School of Medicine, Tsu, Mie, Japan; 4 Mie University Medical Zebrafish Research Center, Tsu, Mie, Japan; 5 Department of Bioinformatics, Mie University Life Science Research Center, Tsu, Mie, Japan; 6 Department of Omics Medicine, Mie University Industrial Technology Innovation Institute, Tsu, Mie, Japan; 7 Department of Neurology, Kyoto University Graduate School of Medicine, Kyoto, Japan; 8 Research Team for Mechanism of Aging, Tokyo Metropolitan Institute of Gerontology, Tokyo 173-0015, Japan

## Abstract

Parkin, a ubiquitin E3 ligase of the ring between ring fingers family, has been implicated in mitochondrial quality control. A series of recent reports have suggested that the recruitment of parkin is regulated by phosphorylation. However, the molecular mechanism that activates parkin to induce mitochondrial degradation is not well understood. Here, and in contrast to previous reports that S-nitrosylation of parkin is exclusively inhibitory, we identify a previously unrecognized site of S-nitrosylation in parkin (Cys323) that induces mitochondrial degradation. We demonstrate that endogenous S-nitrosylation of parkin is in fact responsible for activation of its E3 ligase activity to induce aggregation and degradation. We further demonstrate that mitochondrial uncoupling agents result in denitrosylation of parkin, and that prevention of denitrosylation restores mitochondrial degradation. Our data indicates that NO both positive effects on mitochondrial quality control, and suggest that targeted S-nitrosylation could provide a novel therapeutic strategy against Parkinson's disease.

Mutations in the parkin gene, which encodes a ring between ring fingers (RBR) E3 ubiquitin ligase with a ubiquitin-like domain at the N-terminus, are known to cause autosomal recessive juvenile parkinsonism (ARJP)^1^ Recently, a series of cell biological studies have provided strong evidence that parkin plays an important role in maintaining mitochondrial homeostasis^2^. Upon mitochondrial depolarization, parkin is recruited from the cytosol to the mitochondria, where it gets activated, which then promotes mitochondrial degradation via an autophagic event known as mitophagy, triggered by ubiquitination of proteins on mitochondrial outer membrane^3^,^4^.

Recruitment of parkin to the mitochondria is regulated by phosphorylation^5^. PINK1, a serine/threonine kinase with a predicted mitochondrial target sequence and a putative transmembrane domain at the N-terminus, is constitutively proteolysed at the mitochondrial membrane of healthy mitochondria. PINK1 accumulates in the mitochondria and parkin is phosphorylated at Ser65 in a PINK1-dependent manner, leading to its recruitment to the mitochondria^6^,^7^.

The mechanism of recruitment of parkin is well investigated; however, the mechanism of activation of parkin is not known. We hypothesized that the activity of parkin might be regulated by S-nitrosylation, the covalent incorporation of a nitric oxide (NO) moiety into thiol groups. Although it has been shown that S-nitrosylation is enhanced in brain tissues of patients with PD and in cultured cells treated with rotenone, an inhibitor of the mitochondrial respiratory chain complex I, physiological or pathophysiological significance of these observations is still controversial^8^,^9^.

In this study, we have identified a single cysteine residue in parkin that was predominantly S-nitrosylated. To clarify the role of S-nitrosylation in regulating the activity of parkin and subsequent mitochondrial degradation, we created mutants by replacing this cysteine residue with other amino acids and then evaluated the effect of S-nitrosylation on parkin's function by comparing the activities of the mutant and wild-type parkins.

## Results

### S-nitrosylation of parkin increases its E3 ligase activity upon mitochondrial depolarization

**Figure 1:**
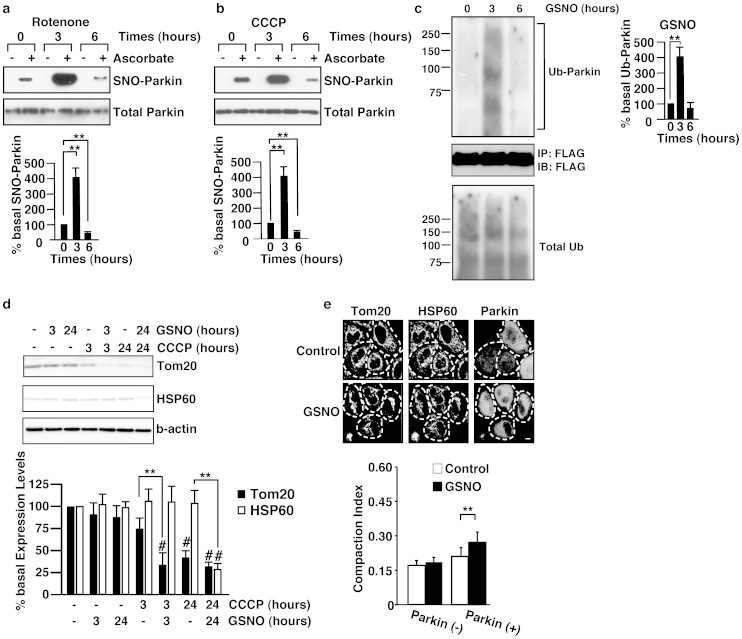
S-nitrosylation of parkin enhances the E3 ligase activity and mitochondrial degradation. SH-SY5Y cells transfected with the FLAG-tagged wild-type parkin plasmid were treated with 1 μM rotenone (a) or 10 μM CCCP (b) for the indicated times, and cell lysates were analysed by SNO-RAC, in which ascorbate-dependent purification demonstrates the presence of S-nitrosylated cysteine residues, followed by immunoblot analysis with anti-parkin antibody (upper panels). The quantity of S-nitrosylated parkin, as measured by scanning densitometry, is expressed as a percentage of control, normalized with respect to total parkin. Data are means ± SE (n = 4); **p < 0.01 versus control (lower panels). (c) SH-SY5Y cells were transfected with the FLAG-tagged parkin plasmid and treated with GSNO (50 μM) for the indicated times. Lysates were used for immunoprecipitation with anti-FLAG antibody, and immunoprecipitates were immunoblotted with anti-ubiquitin (upper panel) or anti-FLAG (middle panel) antibody and lysates were immunoblotted with anti-ubiquitin as control (lower panel). (d) Lysates prepared from HeLa cells treated with 50 μM GSNO and/or 10 μM CCCP for different time periods as indicated were immunoblotted with anti-Tom20, anti-HSP60 or anti-beta-actin antibodies (upper panel). The quantity of Tom20 and HSP60, as measured by scanning densitometry, is expressed as a percentage of control, normalized with respect to beta-actin (lower panel). Data shown are mean ± SE (n = 4); **p < 0.01 versus GSNO-untreated samples, and # means p < 0.01 versus control. (e) HeLa cells transfected with the Venus-tagged parkin expression plasmid were incubated with 50 μM GSNO for 3 hr, and then immunostained with anti-Tom20 and anti-HSP60 antibodies (upper panel). The dotted line surrounded a parkin-expressing cell. The compaction index was calculated from the images stained with anti-Tom20 antibody as described in Materials and Methods (lower panel). Data shown are mean ± SE (n = 15); **p < 0.01 versus GSNO-untreated samples. Scale bars in images = 10 μm. Full scans of the blots in a, b and d are available in Supplementary Information, Fig.S10.

To investigate the role of S-nitrosylation on parkin's function in mitochondrial degradation, we first examined the level of S-nitrosylation in parkin using the modified biotin-switch assay for protein S-nitrosothiols, using resin-assisted capture (SNO-RAC)^10^. S-nitrosylation of parkin increased when treated for 3 hr with either the mitochondrial Complex I inhibitor rotenone or the mitochondrial-uncoupling reagent carbonylcyanide m-chlorophenylhydrazone (CCCP); however, longer treatment with rotenone or CCCP resulted in marked denitrosylation of parkin (Figs. 1a and b). We analysed the E3 ligase activity of parkin by measuring autoubiquitination, and found that treatment with S-nitrosoglutathione (GSNO), rotenone or CCCP for 3 hr stimulated the activity of parkin and longer treatment with GSNO or rotenone brought the activity back to the basal level (Fig. 1c and Supplemental Fig. S1). To test whether S-nitrosylation regulates mitochondrial degradation, HeLa cells, which expressed negligible amount of endogenous parkin (Supplementary Fig. S2), were transfected with the FLAG-tagged wild-type parkin expression plasmid, treated with CCCP in the absence or presence of GSNO, and then the cells were analysed by immunoblot for Translocase of outer membrane 20 (Tom20) and Heat shock protein 60 (HSP60), proteins found in the outer membrane and matrix of mitochondria, respectively. GSNO reduced the level of Tom20 at 3 hr and that of HSP60 at 24 hr after the treatment of CCCP (Fig. 1d), indicating that GSNO stimulates the CCCP-mediated degradation of mitochondria. Shortly following mitochondrial depolarization, parkin induces aggregation and perinuclear localization of mitochondria^11^. Thus, we adopted the compaction index to obtain a quantitative measure of the relative mitochondrial aggregation^12^. Interestingly, GSNO induced aggregation of mitochondria in HeLa cells overexpressing the parkin without recruiting parkin to the mitochondria, but GSNO did not induce such mitochondrial aggregation in HeLa cells not expressing parkin (Fig. 1e). These data suggested that S-nitrosylation might regulate mitochondrial quality control via activation of parkin.

### Identification of an S-nitrosylated cysteine residue in parkin

**Figure 2:**
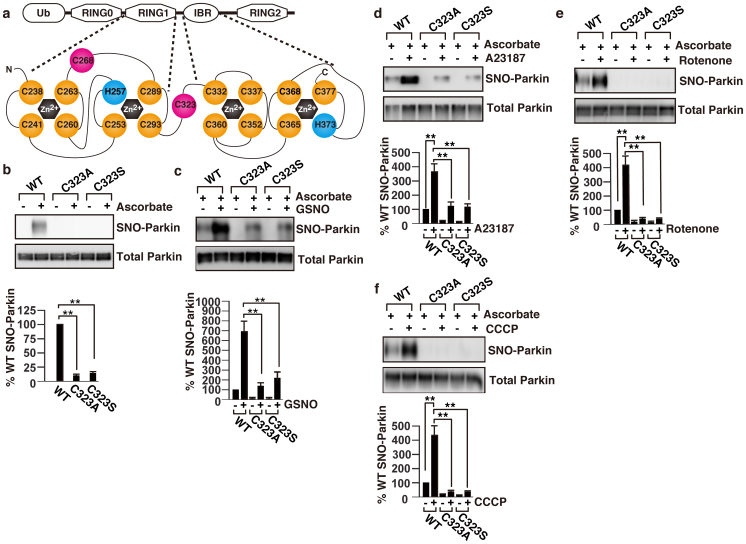
Cys323 is the critical cysteine residue that is S-nitrosylated in parkin. (a) A schematic representation of the structure of the RING1 and IBR domains in parkin. Yellow and red circles indicate cysteines involved in zinc coordination and not involved in zinc coordination, respectively. Blue circle indicates histidine involved in zinc coordination. (b) Cell lysates from SH-SY5Y cells transfected with the FLAG-tagged wild type, C323A or C323S parkin expression plasmid were analysed by SNO-RAC as above (upper panel). The quantity of S-nitrosylated parkin, as measured by scanning densitometry, is expressed as a percentage of control, normalized with respect to total parkin. Data shown are mean ± SE (n = 4); **p < 0.01 versus wild type (lower panel). SH-SY5Y cells transfected with the FLAG-tagged wild type, C323A or C323S parkin expression plasmid were incubated for 3 hr with 50 μM GSNO (c), 8 μM A23187 (d), 1 μM rotenone (e), or 10 μM CCCP (f), and then the cell lysates were analysed by SNO-RAC (upper panels). The quantity of S-nitrosylated parkin, as measured by scanning densitometry, is expressed as a percentage of control, normalized with respect to total parkin. Data shown are mean ± SE (n = 3); **p < 0.01 (lower panels). Full scans of the blots in b, c, d, e and f are available in Supplementary Information, Fig.S10.

Parkin is a 465-residue protein comprising of an N-terminal ubiquitin-like domain, a unique parkin-specific domain in the central region, and two RING finger domains (RING1 and RING2) separated by an in-between ring (IBR) domain (Fig. 2a)^13^. To determine the site at which parkin is S-nitrosylated, we performed domain S-nitrosylation mapping studies using truncated mutants of parkin. Consistent with the previous report^8^, results of our mapping studies indicated that the S-nitrosylated cysteine residue(s) is(are) located in the RING1 domain and/or in the IBR domain of parkin (Supplemental Figs. S3a and S3b).

The architecture of the cysteine and histidine rich RBR domain is highly conserved. The RING domain binds two zinc ions in a cross-brace fashion using a C3HC4 motif. In case of parkin, Cys238, Cys241, Cys253, His257, Cys260, Cys263, Cys289, and Cys293 residues of RING1 domain are involved in zinc coordination (Fig. 2a)^13^. The IBR domain is different from the RING finger domain, but also binds two zinc ions using residues Cys332, Cys337, Cys352, Cys360, Cys365, Cys368, His373, and Cys377 (Fig. 2a)^14^. We hypothesized that the critical cysteine that is S-nitrosylated, which causes activation of parkin, is not involved in zinc coordination. To test this hypothesis, we generated individual parkin mutants by site-directed mutagenesis and converted each cysteine residue to an alanine or a serine residue. S-nitrosylation assay using SNO-RAC resin showed no detectable level of S-nitrosylation in C323A and C323S mutants (Fig. 2b and Supplemental Fig. S3c). The S-nitrosylation levels of all other cysteine mutants and disease related mutants of parkin were similar to that of the wild-type (Supplemental Figs. S3c and S3d).

We next examined whether the wild-type, C323A and C323S parkins could be S-nitrosylated using exogenous NO donor. Treatment with 50 μM GSNO increased S-nitrosylation of the wild type and C323S mutant, but the level of S-nitrosylated C323S parkin was significantly less than that of the wild type (Fig. 2c). Treatment with A23187, which activates nNOS, showed similar trends in S-nitrosylation of the wild-type and C323S mutant as the GSNO (Fig. 2d). Furthermore, levels of S-nitrosylated C323S parkin were negligible when treated with rotenone or CCCP (Figs. 2e and 2f, and Supplemental Fig. S3e). Taken together, these results suggested that Cys323 is the critical cysteine which is S-nitrosylated, and exogenous NO or calcium overlord could cause S-nitrosylaion of other cysteine(s), but to a lesser extent than the Cys323.

### S-nitrosylation increases E3 ligase activity of parkin

**Figure 3:**
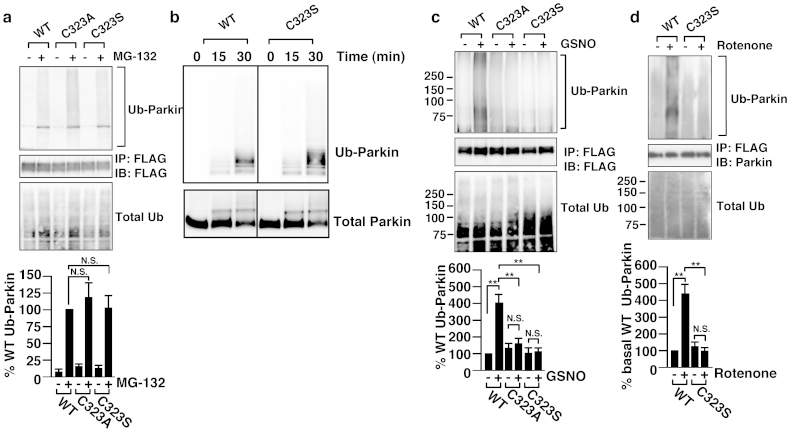
Effect of S-nitrosylation on the activity of parkin. (a) SH-SY5Y cells were transfected with the wild-type or mutants parkin and treated with 10 μM MG132 for 3 hr. Lysates were immunoprecipitated with anti-FLAG antibody, and immunoprecipitates were immunoblotted with anti-ubiquitin or anti-FLAG antibody and lysates were with anti-ubiquitin as control as indicated. The quantity of ubiquitinated parkin, as measured by scanning densitometry, is expressed as a percentage of wild type treated with MG132, normalized with respect to total parkin and ubiquitinated proteins. Data shown are mean ± SE (n = 4); N.S. means no significant difference versus the wild-type. (b) E3 ligase activity of parkin was analysed in vitro using wild-type and C323S parkins purified from HEK293 cells. Immnoblot analyses were performed using anti-ubiquitin antibody (Ub-parkin) or anti-FLAG antibody (Total parkin). (c) SH-SY5Y cells were transfected with the FLAG-tagged wild-type or mutants parkin and treated with 50 μM GSNO for 3 hr in the presence of 10 μM MG132. Lysates were immunoprecipitated with anti-FLAG antibody, and immunoprecipitates were immunoblotted with anti-ubiquitin or anti-FLAG antibody, and lysates were with anti-ubiquitin as control as indicated. The quantity of ubiquitinated parkin, as measured by scanning densitometry, is expressed as a percentage of wild-type without GSNO treatment, normalized with respect to total parkin and ubiquitinated proteins. Data shown are mean ± SE (n = 4); **p < 0.01 and N.S. means no significant difference. (d) SH-SY5Y cells were transfected with the wild-type or C323S parkin and treated with 1 μM rotenone and 10 μM MG132 for 3 hr. Lysates were immunoprecipitated with anti-FLAG antibody, and immunoprecipitates were immunoblotted with anti-ubiquitin or anti-FLAG antibody, and lysates were with anti-ubiquitin as control as indicated. The quantity of ubiquitinated parkin, as measured by scanning densitometry, is expressed as a percentage of wild-type without rotenone treatment, normalized with respect to total parkin and ubiquitinated proteins. Data shown are mean ± SE (n = 4); **p < 0.01 and N.S. mean significant and no significant difference, respectively. Full scans of the blots in b are available in Supplementary Information, Fig.S10.

We next determined whether S-nitrosylation of Cys323 has any effect on the ubiquitin E3 ligase activity of parkin. Treatment with a proteasome inhibitor, N-(benzyloxycarbonyl)leucinylleucinylleucinalZ-Leu-Leu-Leu-al (MG132), increased the autoubiquitination of the wild-type, C323A and C323S parkins (Fig. 3a), indicating that the E3 ligase activities of both cysteine mutants were similar to that of the wild type under the basal condition, and this observation was further confirmed by using an in vitro ubiquitination assay (Fig. 3b and Supplemental Figure S4a and S4b). We next determined whether S-nitrosylation modulates the ubiquitin E3 ligase activity of parkin. As shown in Fig. 3c, GSNO treatment significantly stimulated autoubiquitination of the wild-type parkin but not that of the C323A and C323S parkin mutants. To further confirm the importance of Cys323 on parkin's E3 ligase activity, we examined the autoubiquitination levels of parkin following treatment with rotenone. As shown, rotenone treatment increased the autoubiquitination of the wild-type parkin but not that of the C323S (Fig. 3d). Taken together, these results suggested that Cys323 plays a critical role in regulating the E3 ligase activity of parkin by S-nitrosylation.

### S-nitrosylation increases mitochondrial degradation by depolarization

**Figure 4:**
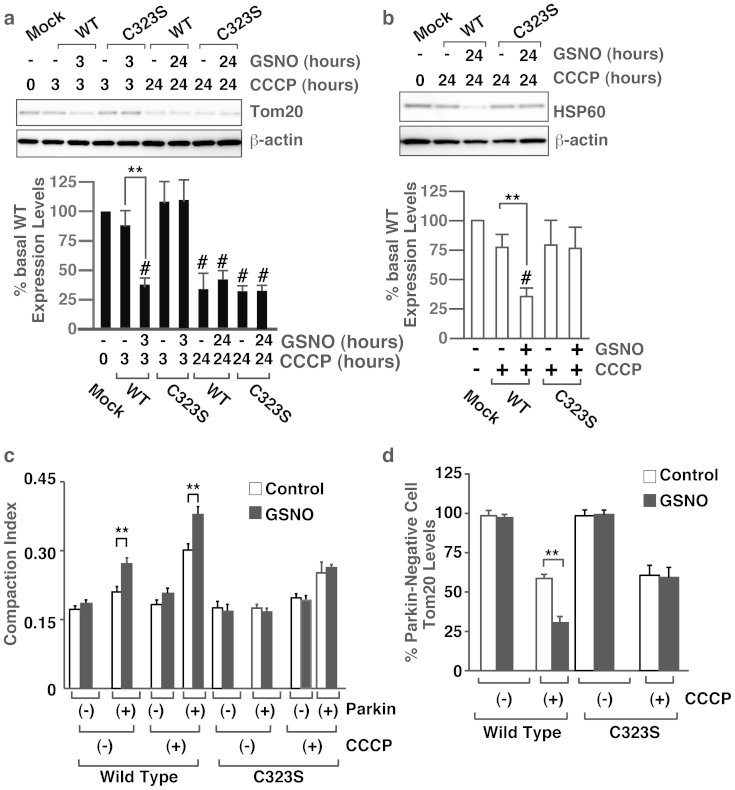
S-nitrosylation of Cys323 in parkin by exogenous NO regulates mitochondrial degradation by membrane potential depolarization. (a) and (b) Lysates prepared from HeLa cells treated with GSNO (50 μM) and/or CCCP (10 μM) for different time periods as indicated were immunoblotted with anti-Tom20, anti-HSP60 and anti-beta-actin antibodies (upper panel). The quantity of Tom20 (a) and HSP60 (b), as measured by scanning densitometry, is expressed as a percentage of control, normalized with respect to beta-actin (lower panel). Data shown are mean ± SE (n = 4); **p < 0.01 versus GSNO-untreated samples, and # means p < 0.01 versus control. (c) and (d) HeLa cells transfected with the Venus-tagged parkin expression plasmid were incubated with GSNO (50 μM) and/or CCCP (10 μM) for different time periods as indicated, and then immunostained with anti-Tom20 antibody. The compaction index was calculated from the images stained with anti-Tom20 antibody as described in Materials and Methods (c). The data used for the wild-type cells without CCCP was the same data as in Figure 1e. Data shown are mean ± SE (n = 15); **p < 0.01 versus GSNO-untreated samples. The quantity of Tom20, as measured from scanned images using *ImageJ (NIH)*, is expressed as a percentage of parkin-negative cells (d). Data shown are mean ± SE (n = 10); **p < 0.01 versus GSNO-untreated cells. Full scans of the blots in a and b are available in Supplementary Information, Fig.S10.

**Figure 5:**
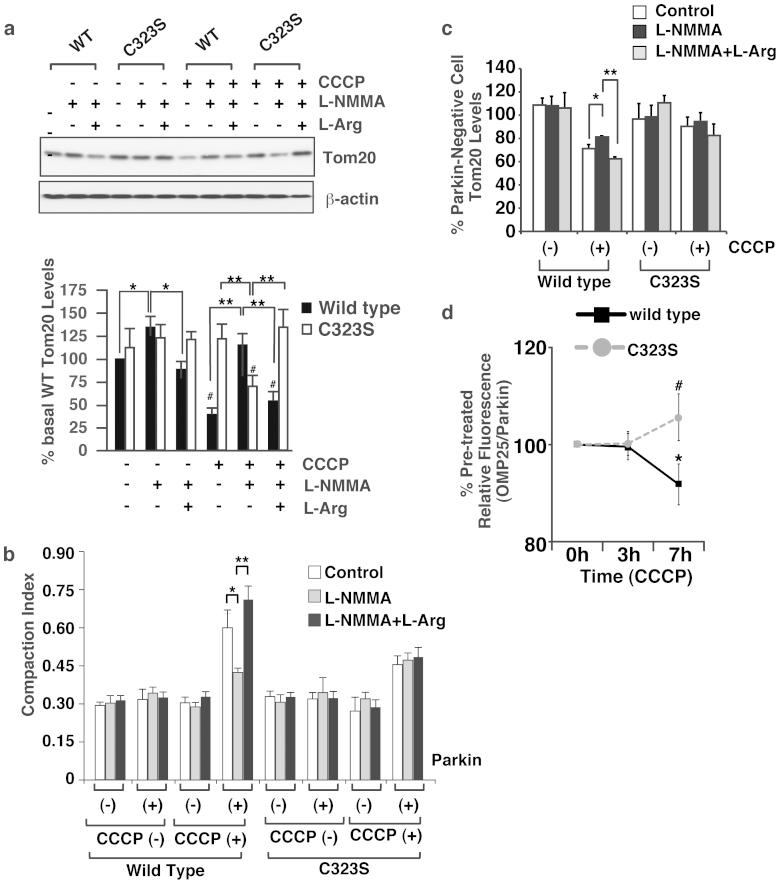
S-nitrosylation of Cys323 in parkin by endogenous NO regulates mitochondrial degradation by membrane potential depolarization. (a) After pre-incubation with L-NMMA (0.5 mM) and/or L-arginine (1.0 mM) in serum-free medium for 1 hr, HeLa cells were treated with CCCP (10 μM), L-NMMA (0.5 mM) and/or L-arginine (1.0 mM) in serum-free medium for 3 hr, and then lysates were immunoblotted with anti-Tom20 and anti-beta-actin antibodies (upper panel). The quantity of Tom20, as measured by scanning densitometry, is expressed as a percentage of control, normalized with respect to beta-actin (lower panel). Data shown are mean ± SE (n = 3); *p < 0.05 and **p < 0.01. (b) and (c) After pre-incubation with L-NMMA (0.5 mM) and/or L-arginine (1.0 mM) in serum-free medium for 1 hr, HeLa cells transfected with the Venus-tagged parkin expression plasmid were treated with CCCP (10 μM), L-NMMA (0.5 mM) and/or L-arginine (1.0 mM) in serum-free medium for 3 hr, and then immunostained with anti-Tom20 antibody. The compaction index was calculated as described above (b). Data shown are mean ± SE (n = 5); **p < 0.01 versus control. The quantity of Tom20, as measured from scanned images using *ImageJ (NIH)*, is expressed as a percentage of parkin-negative cells (c). Data are means ± SE (n = 4); *< 0.05 and **p < 0.01. (d) At 48 hpf, zebrafish larva expressing EGFP-OMP25 and Parkin (wild type or C323S) were treated with 300 nM CCCP for 3 and 7 hours. In vivo fluorescence imaging was performed at pre-, 3-hours and 7-hours treatment of CCCP. The ratio of fluorescent intensity between EGFP-OMP and mCherry-Parkin was calculated and expressed as a percentage of that of pre-treatment of CCCP in each zebrafish. Data are means ± SE (n = 5 for wild-type Parkin and n = 9 for C323S Parkin). * p < 0.05 versus the pre-treatment of CCCP. # means p < 0.05 versus wild-type parkin. Full scans of the blots in a are available in Supplementary Information, Fig.S10.

We next evaluated the effect of S-nitrosylation on the degradation of mitochondria using parkin expressing HeLa cells, in which we have also observed activation of pakin in GSNO-dependent manner (Supplemental Figure S4). As described above (Fig. 1d), GSNO increased the degradation of Tom20 and HSP60 in HeLa cells overexpressing the wild-type parkin, but not in cells overexpressing the C323S mutant (Figs. 4a and 4b). Immunostaining experiments showed that GSNO had no effect on the aggregation and degradation of mitochondria in C323S-overexpressing cells (Figs. 4c and 4d, and Supplemental Fig. S5a). To assess the effect of endogenous NO produced by nitric oxide synthase (NOS), we examined the aggregation and degradation of mitochondria following treatment with NOS inhibitor L-NMMA. As shown in Fig. 4a, L-NMMA treatment of HeLa cells overexpressing the wild-type parkin led to increased expression of Tom20 even in the presence of CCCP and addition of L-arginine reversed the effect, but this increase in Tom20 expression was not observed in HeLa cells overexpressing the C323S mutant (Fig. 5a). Immunostaining experiments showed that L-NMMA treatment decreased the mitochondrial aggregation and degradation in the wild-type parkin expressing HeLa cells and L-Arginine reversed the effect of L-NMMA, and that L-NMMA and L-Arginine had no effect on the mitochondrial aggregation and degradation in C323S-overexpressing cells (Figs. 5b and 5c, and Supplemental Fig. S5b). Furthermore, we evaluated mitochondrial degradation by depolarization in vivo using zebrafishes expressing EGFP localized at mitochondrial outer membrane (EGFP-OMP25)^4^ and parkin fused to a fluorescent protein, mCherry. Treatment of CCCP for 7 hours significantly decreased the relative fluorescence of EGFP-OMP25 in zebrafishes overexpressing wild-type Parkin but not in zebrafishes overexpressing C323S mutant (Fig. 5d). In addition, to eliminate the possibility that the S-nitrosylation of parkin may increase the mitochondrial degradation by regulating the phosphorylation and subsequent recruitment of parkin, we assessed the phosphorylation and recruitment of wild-type and C323S mutant of parkin following the treatment of respective cells with CCCP and found no apparent differences (Supplemental Fig. S6). Taken together, these results suggested that NO regulates the mitochondrial aggregation and degradation via S-nitrosylation of Cys323 in parkin by activating its E3 ligase activity.

### Peroxynitrite negatively regulates mitochondrial degradation

**Figure 6:**
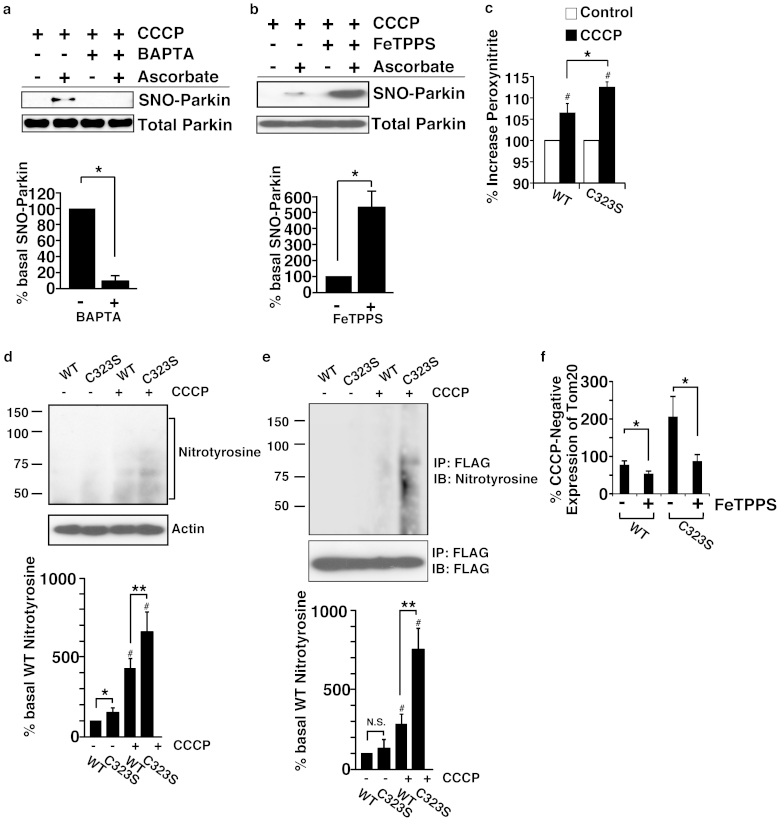
Peroxynitrite negatively regulates mitochondrial degradation. (a) and (b) SH-SY5Y cells overexpressing parkin were treated with BAPTA-AM (10 μM) (a) or FeTPPS (5 μM) (b) with CCCP (10 μM) for 3 hr, and lysates were analysed by SNO-RAC. The quantity of S-nitrosylated parkin, as measured by scanning densitometry, is expressed as a percentage of control, normalized with respect to total parkin. Data shown are mean ± SE (n = 3); *p < 0.05 and **p < 0.01 versus control respectively. (c) SH-SY5Y cells overexpressing wild-type or C323S parkin were incubated with CCCP (10 μM) for 6 hr and the peroxynitrite was measured by flow cytometry using APF (10 μM). Increases in peroxynitrite by CCCP were calculated as relative fold-changes in fluorescence compared to the untreated control. Data shown are mean ± SE (n = 4); *p < 0.05 versus wild type. (d) and (e) SH-SY5Y cells overexpressing wild-type or C323S parkin were incubated with CCCP (10 μM) for 3 hr. Cell lysates were immunoblotted with anti-nitrotyrosine and anti-beta-actin antibodies (d), or immunoprecipitated with anti-FLAG antibody and the immunoprecipitates were immunoblotted with anti-nitrotyrosine and anti-FLAG antibodies (e). The quantity of nitrotyrosine, as measured by scanning densitometry, is expressed as a percentage of CCCP-untreated wild-type, normalized with respect to beta-actin (d) or FLAG (e). Data shown are mean ± SE (n = 3); *p < 0.05, **p < 0.01 and N.S. represent significant and no significant difference versus the wild-type, respectively. # means p < 0.01 versus CCCP-untreated. (f) After preincubation with FeTPPS (5 μM), SH-SY5Y cells were treated with CCCP (10 μM) and FeTPPS (5 μM) for 3 hr, and then lysates were immunoblotted with anti-Tom20 and anti-beta-actin antibodies. Decreases in the level of Tom20 by CCCP were calculated as relative fold-changes in signal compared to the CCCP-untreated control, normalized with respect to beta-actin. Data shown are mean ± SE (n = 8); *p < 0.05 versus FeTPPS-untreated. Full scans of the blots in a, b, d and e are available in Supplementary Information, Fig.S10.

To further investigate the mechanism of S-nitrosylation of parkin by mitochondrial membrane potential depolarization, we measured cytosolic calcium levels in SH-SY5Y cells treated with rotenone and CCCP. Both rotenone and CCCP increased the cytosolic calcium level (Supplemental Figs. S7a and S7b) and calcium chelator 1,2-bis(o-aminophenoxy)ethane-N,N,N′,N′-tetraacetic acid (BAPTA) decreased both production of NO (Supplemental Fig. S7c) and S-nitrosylation of Parkin (Fig. 6a) induced by CCCP. Since it was reported earlier that peroxynitrite increases the S-nitrosylation of proteins^15^, we therefore examined the effect of sodium tetra-p-sulfophenylporphine iron (III) (FeTPPS), a peroxynitrite decomposition catalyst, on the S-nitrosylation of parkin in SH-SY5Y cells. Contrary to our expectation, FeTPPS increased S-nitrosylation of parkin, indicating that peroxynitrite decreased S-nitrosylation of parkin (Fig. 6b). To investigate the effect of peroxynitrite on mitochondrial degradation, we determined the level of peroxynitrite and nitrotyrosine, which is a product of tyrosine nitration generated by reactive nitrogen species (RNS), such as peroxynitrite^16^. Level of peroxynitrite and 3-nitrotyrosine in the C323S-expressing cells was higher than that in the wild-type-expressing cells (Figs. 6c, 6d and Supplemental Fig. S8), suggesting that the impairment of mitochondrial degradation might enhance the production of peroxynitrite. Furthermore, wild-type and C323S parkins were modified by peroxynitrite to generate 3-nitrotyrosine and the modification of C323S was more enhanced than the wild-type (Fig. 6e). Finally, to determine whether peroxynitrite has any effect on mitochondrial degradation, we incubated wild-type and C323S expressing SH-SY5Y cells with CCCP in the presence of FeTPPS and then assessed the levels of Tom20 in these cells by immunoblot. As shown in Fig. 6f, FeTPPS increased the degradation of Tom20 in both wild-type and C323S expressing cells, indicating the presence of a mechanism for inhibiting the mitochondrial degradation by peroxynitrite which, at least partly, is independent of Cys323.

## Discussion

Elucidation of the underlying mechanism that regulated the function of parkin may not only have important implications in understanding the depolarization-dependent degradation of mitochondria but may also lead to the development of a therapeutic method for the treatment of Parkinson's disease. In this report, we have identified a cysteine residue in parkin that is S-nitrosylated, and showed that S-nitrosylation upregulates the E3 ligase activity of parkin and subsequent mitochondrial degradation induced by mitochondrial depolarization.

Parkin has the canonical RING1 domain, which provides the E2 ubiquitin-conjugating enzyme-binding region, and the non-canonical RING2 domain, which contains the cysteine that receives the ubiquitin from E2 (Fig. 2a)^10^. The structure of the IBR domain suggests that this domain orients the adjacent RING1 and RING2 domains to regulate ubiquitination^17^. In this study, we showed that the Cys323 in the IBR domain, a cysteine residue that is not involved in zinc ion coordination, was predominantly S-nitrosylated. We then generated C323A and C323S mutants and showed that these mutants retained the E3 ligase activity (Figs. 3a and 3b), indicating that the basal activity of parkin was preserved in the Cys323 to alanine and in the Cys323 to serine mutants. On the other hand, mutants of Cys289 and Cys418, which are involved in zinc ion coordination in RING1 and RING2, respectively, were reported to have lost the activity and cause ARJP^18^. Furthermore, treatment with GSNO or rotenone led to increased autoubiquitination of the wild-type parkin but not that of the C323S mutant of parkin (Figs. 3c and 3d), suggesting that NO regulates parkin's activity via the Cys323 residue. Taken together, our data showed that S-nitrosylation of Cys323 regulates the activity of parkin, keeping the zinc-associating structure of IBR domain in place, possibly by bringing the RING1 and RING2 domains within close proximity of each other.

Although S-nitrosylation of parkin was reported earlier by two groups^8^,^9^, they however reached to different conclusions than ours. Dawson's group claimed that S-nitrosylation of parkin had reduced E3 ligase activity 6 hr after the treatment of NO-donor^8^, whereas we observed that S-nitrosylation of parkin increased 3 hr after the treatment. This apparent discrepancy could be explained by considering the time-dependent regulation of S-nitrosylation and denitrosylation: parkin is S-nitrosylated after 3 hr of treatment with rotenone or CCCP, but it gets denitrosylated after 6 hr of treatment (Figs. 1a and 1b, and Supplemental Fig. S3e), when the S-nitrosylation-mediated stimulation of the E3 ligase activity should be dissolved (Fig. 1a). Lipton's group showed that multiple S-nitrosylated cysteine residues existed in the RING1 domain of parkin^9^, whereas we showed that only the Cys323 in the IBR domain of parkin was S-nitrosylated. Lipton's group also claimed that S-nitrosylation increases parkin's activity within the first few hours. Most cysteine residues in the RING1 domain are, however, involved in zinc coordination and S-nitrosylation of the cysteine residues should, therefore, disrupt the conformation of RING1, leading to inactivation of parkin. As pointed out above, Cys323 is not involved in zinc coordination, and therefore its modification could regulate the activity of parkin without disrupting the conformation of the IBR domain. We have discussed the technical differences leading to the discrepancies in detail in the Supplemental Discussion.

The function of parkin is also regulated by phosphorylation and recruitment of parkin^2^. To clarify if there is any link between S-nitrosylation and phosphorylation of parkin, we have performed several experiments. First, localization of Venus-tagged parkin, as investigated by immunofluorescence, showed that GSNO had no effect on the recruitment of parkin to the mitochondria, even though this treatment induced mitochondrial aggregation (Fig. 1e). Second, we compared the CCCP-induced phosphorylation and recruitment of the wild-type and C323S parkins and found no differences (Supplemental Fig. S6). Third, the disease-related mutants of parkin, K161N and K211N, mitochondrial localization of which are severely compromised, were S-nitrosylated as well as the wild type (Supplemental Fig. S3d), suggesting that parkin is S-nitrosylated in the cytosol. Taken together, our results suggested that S-nitrosylation and phosphorylation regulate parkin's function independently (Supplemental Fig. S9).

Although the main function of parkin is linked to its E3 ligase activity, Checler's group showed parkin also functions as a p53 transcriptional repressor in an E3 ligase-independent manner^19^. In this report, we showed S-nitrosylation of parkin increases E3 ligase activity, which leads to the conclusion that S-nitrosylation should not affect the transcriptional function of parkin. However, they showed the disease-related mutations of parkin impair the transcriptional activity, indicating the disease-related mutations might disrupt the conformation of RING1-IBR-RING2 domains which affect the transcriptional activity of parkin. In this context, S-nitrosylation could regulate the transcriptional activity of parkin by altering the conformation, in addition to E3 ligase activity.

Production of NO could have two-sided effects on the mitochondrial quality control. Though we showed that S-nitrosylation stimulates the E3-ligase activity of parkin and mitophagy, which should have protective effect, NO is also known to exacerbate neurodegeneration induced by 1-Methyl-4-phenyl-1,2,3,6-tetrahydropyridine (MPTP), a mitochondrial complex I inhibitor^20^,^21^. Transient and partial mitochondrial dysfunction could be recovered by S-nitrosylation-mediated enhanced disposal of defective mitochondria. On the other hand, mitochondrial dysfunction could be beyond the disposal capacity, causing the mitochondria to be disposed to remain, which would lead to the production of reactive oxygen species (ROS), including the superoxide^16^. Mitochondrial superoxide reacts with NO forming the potent oxidant peroxynitrite, which promotes cellular damages. We showed that peroxynitrite denitrosylated parkin (Fig. 6b) and increased tyrosine nitration of parkin (Fig. 6d) in SH-SY5Y cells treated with CCCP. We also demonstrated that FeTPPS increased the mitochondrial degradation in cells expressing the wild-type or the C323S parkin (Fig. 6f), suggesting that tyrosine nitration might be involved in inhibiting the peroxynitrite-mediated degradation of mitochondria. Taken together, these results suggest that even though NO activates parkin via S-nitrosylatlion, it decreases mitochondrial degradation by producing peroxynitrite, which inhibits the protective effect of NO (Supplemental Figure S9).

Based on our findings, we suggest that the dynamic S-nitrosylation of parkin exerts a protective effect on the mitochondrial degradation, which in turn can protect the cells from mitochondrial dysfunction. Parkin plays an important role in mitochondrial degradation by ubiquitination of mitochondrial membrane proteins. Moreover, parkin mutants from ARJP patients exhibited loss of E3 ligase activity^1^,^18^, suggesting that activation of parkin might have a therapeutic effect on sporadic PD. Our findings support the idea that activation of parkin by S-nitrosylation might provide a novel therapeutic modality. However, as NO could exacerbate neurodegeneration via production of peroxynitrite, development of tools to upregulate S-nitrosylation of parkin without the production of peroxynitrite might be required to successfully achieve this goal.

## Methods

### Materials

Anti-FLAG monoclonal antibody, CCCP and L-NMMA were obtained from Sigma-Aldrich (St. Louis, MO). Anti-nNOS, anti-ubiquitin and anti-Tom20 antibodies were purchased from Santa Cruz (Santa Cruz, CA). Anti-HSP60 antibody was from BD Transduction Laboratories (San Jose, CA). Rotenone was obtained from Nacalai Tesque (Kyoto, Japan) and Phos-tag™ gel was obtained form Wako Pure Chemical Industries (Osaka, Japan). BAPTA/AM and FeTPPS were obtained from Merck-Millipore (Darmstadt, Germany). ARF and DAF-FM DA were obtained from Sekisui medical (Tokyo, Japan).

### Construction of plasmids

To construct plasmids for overexpressing tagged parkin, the coding region of parkin was amplified from the human cDNA by Polymerase chain reaction (PCR) and inserted into pcDNA3.1 for tagging with a FLAG tag or inserted into pcDNA3 for tagging with Venus, a modified yellow fluorescent protein. Single cysteine mutants and truncated forms of parkin were generated using the PrimeSTAR® Mutagenesis Basal Kit (Takara, Otsu, Shiga, Japan) and appropriate primers following manufacturer's instructions. The disease-related mutants of Parkin were gifts from Drs. N Matsuda and K. Tanaka (Tokyo Metropolitan Institute of Medical Science)^22^.

### Cell culture and transfection

Cells were maintained in Dulbecco's modified Eagle's medium supplemented with 10% fetal bovine serum (FBS), 100 U/ml penicillin and 100 μg/ml streptomycin at 37°C in a humidified 5% CO2 atmosphere. Cells were transfected at 80% confluency using LipofectAMINE LTX (Invitrogen: Carlsbad, CA) for SH-SY5Y cells and DreamFect™ Gold (OZ BIOSCIENCES, France) for HeLa cells and following manufacturer's instructions.

### Detection of S-nitrosylated proteins by SNO-RAC

S-nitrosylated proteins were detected by using the SNO-RAC method as described^9^ with some modifications. Briefly, SNO-RAC resins were prepared as described. 250 μL of cell lysates were diluted with 750 μL of HEN buffer [250 mM HEPES, 1 mM EDTA, 0.1 mM neocuproine, pH 8.0] and incubated with 1% SDS (final concentration) and 0.1% methyl methanethiosulfonate (Sigma-Aldrich, St. Louis, MO) at 50°C for 25 min. Proteins were precipitated with acetone, washed three times with 70% acetone and resuspended in 200 μL HENS buffer (HEN containing 1% SDS). This was added to 50 μL resin slurry in the presence of sodium ascorbate (Fluka, final 20 mM), mixed by rotation in the dark for 3 hr, following which the resin was washed with 4 × 1 mL HENS buffer. Captured proteins were eluted with 30 μL HENS buffer containing 100 mM 2-mercaptoethanol for 20 min at RT, and 20 μL of each eluent was used for SDS-PAGE analysis.

### In vivo ubiquitination assay

Cells transfected with the FLAG-tagged wild-type, Cys323A or Cys323S parkin expression plasmid were treated with the indicated drug as described previously^8^ with some modification. After incubation with a given drug, cells were harvested by washing with cold PBS and then lysed using the lysis buffer of Qproteome Mammalian Protein Prep Kit (Qiagen, Germany). The lysates were immunoprecipitated using Dynabeads® Magnetic Beads according to the standard protocol. Proteins in the precipitates and cell lysates were resolved on SDS-PAGE gel and subjected to immunoblot analysis using anti-ubiquitin (for both immunoprecipitates and lysates) or anti-FLAG antibodies (for the precipitates).

### Confocal microscopy

For experiments using L-NMMA and/or L-arginine, we pre-incubated cells with L-NMMA (0.5 mM) and/or L-arginine (1.0 mM) in serum-free medium for 1 hr and then cells were treated with drugs in serum-free medium.

Cells were fixed with 4% paraformaldehyde in PBS and permeabilized with 0.2% Triton X-100 in PBS. Cells stained with appropriate antibodies were imaged using a laser-scanning microscope (FV1000, Olympus, Tokyo, Japan) fitted with UPlanSApo 40×/0.9NA lens (Olympus, Tokyo, Japan). To quantify the compaction index of mitochondria, mid plane images of cells immunostained for Tom20 were obtained as described previously^11^. In ImageJ (NIH), the Tom20 channel was converted into binary and the area and perimeter of mitochondria within the cell of interest (selected using the “region of interest” tool) was measured using the “analyze particles” function. The compaction index (which is the perimeter of a circle with the same area as the object of interest divided by the actual perimeter of the object of interest) was calculated from the perimeter (P) and the area (A) using the following formula: (2π * ((A/π)^1/2^))/P. To quantify the intensities of Tom20 and HSP60, mid plane images of cells immunostained for Tom20 were analysed using ImageJ (NIH) and the degradation of Tom20 was calculated as a percentage of parkin-negative cells in the same images.

### In vitro ubiquitination assay

In vitro ubiquitination assay was performed as described previously^23^ with some modifications. After pre-incubation with 500 ng ubiquitin C (LifeSensors, Malvern, PA), 100 ng E1 (LifeSensors, Malvern, PA), and 500 ng UbcH7 (Sigma-aldrich) in the reaction buffer containing 50 mM Tris-HCl (pH 8.5), 5 mM MgCl_2_, 2 mM DTT, 10 mM ATP at 37°C, parkin (purified from HEK293 cells overexpressing the wild-type or C323S mutant) was added. After incubation at 37°C for 30 min, samples were treated with SDS sample buffer, boiled and proteins were separated by SDS-PAGE. Both polymerized ubiquitin chains and ubiquitinated proteins were detected by immunobloting with anti-ubiquitin and anti-FLAG antibodies.

### Injection of mRNA to express mCherry-Parkin and EGFP-OMP25 in zebrafish

The open reading frames of mCherry and Parkin (wild-type and C323S) were amplified by PCR and cloned in the pCS2P+ (Addgene, Cambridge, MA, USA) using BamHI and BamHI/EcoRI sites, respectively. The DNA coding EGFP and the multiple cloning site fused to the C-terminal of EGFP was amplified using PCR from pEGFP-C2 vector (Clontech, Mountain View, CA, USA). The EGFP-MCS was cloned in the pCS2P+ using ClaI and StuI sites. The cDNA of human OMP25 coding 170–206 residues was amplified by PCR and cloned in the pCS2P-EGFP vector using SacI and EcoRI sites. The sequences of these plasmids were confirmed by DNA sequencing. PCR was performed using SP6 and T3 primers and the cloned pCS2P-mCherry-Parkin (WT or C323S) or pCS2P-EGFP-OMP25 vector as templates. The PCR product was purified using QIAquick PCR purification kit (QIAGEN, Valencia, CA, USA). By using the mMESSAGE mMACHINE SP6 Kit (Life Technologies, Grand Island, NY, USA), mRNAs to express mCherry-Parkin (wild type or C323S) and EGFP-OMP25 were transcribed from the PCR product and purified and concentrated using LiCl precipitation. The RNA concentration was determined using NanoDrop (Thermo Scientific, Wilmington, DE, USA).

Zebrafish (AB line, Zebrafish International Resource Center, Oregon, USA) were bred and maintained according to the methods described by Westerfield with some modification^24^,^25^. Briefly, zebrafish were raised at 28.5 ± 0.5°C with a 14 h/10 h light/dark cycle. We prepared two different mRNA solutions: i) mRNA solution containing 150 ng/μl mCherry-Parkin (wild type) and 100 ng/μl EGFP-OMP25 and ii) mRNA solution containing 150 ng/μl mCherry-Parkin (C323S) and 100 ng/μl EGFP-OMP25. Injection of the mRNA solution was made in the 2–4 cell stages of the zebrafish embryo using a microinjector. Embryos were raised in fish medium (0.07 mM KCl, 2 mM CaCl2, 0.5 mM MgSO4, 0.7 mM NaHCO3, pH 7.4) until 24 hours post fertilization (hpf) and in fish medium containing 200 μM 1-phenyl-2-thiourea to suppress the pigmentation from 24 to 55 hpf.

### In vivo imaging of zebrafish expressing mCherry-Prakin and EGFP-OMP2

At 48 hpf, zebrafish were anesthetized in fish medium containing 2-phenoxyethanol (500 ppm) and transferred onto glass slides. The brains of the zebrafishes were observed using a Zeiss 510 confocal laser scanning microscope (CLSM) using a 10× (NA 0.5) objective lens (Carl Zeiss, Oberkochen, Germany) at a resolution of 512 × 512 pixels and 256-scale (0–255) image. EGFP and mCherry were excited at 488 and 543 nm, respectively. The fluorescence of EGFP and mCherry were detected using a narrow band (500–530 nm) and a broad-band (over 560 nm) path filters, respectively. Z-stacks imaging was performed to cover all brain region expressing mCherry and EGFP. We selected the Z-axis position where the fluorescent signal of EGFP and mCherry was clearly visualized in brain. The fluorescent signal of EGFP and mCherry was quantified using Volocity (Perkin Elmer, Cambridge, MA, USA) as follows. First, the brain region visualized by EGFP and mCherry was manually defined as the region of interest (ROI). Second, the areas showing fluorescent intensity of mCherry from 20 to 254 was detected in the ROI and defined as mCherry area. Third, the area showing fluorescent intensity of EGFP below 255 were detected in the mCherry area and defined as EGFP area. Fourth, the pixel counts were calculated in the mCherry and EGFP areas. Fifth, the pixel counts in the mCherry and EGFP areas were integrated by the mean fluorescent intensity of mCherry and EGFP, respectively. Sixth, the integrated value of EGFP was divided by that of the mCherry to calculate the ratio of fluorescent intensity between OMP and Parkin. After the in vivo fluorescent imaging, zebrafish were treated with CCCP (300 nM). The in vivo fluorescent imaging was also performed after 3 and 7 hours treatment of CCCP.

### Flow cytometry

To measure peroxynitrite using Aminophenyl Fluorescein (APF), SH-SY5Y cells transfected with the wild-type and C323S mutant expression plasmid were incubated with DMEM without phenol red (Invitrogen) at 37°C for 1 hr, and then 10 μM CCCP was added. After incubation at 37°C for 5.5 hr, 10 μM APF was added to the cells and then incubated at 37°C for 30 min. Cells were harvested using cold 1 mL PBS containing 5 mM EDTA, and peroxynitrite was measured by flow cytometry^26^. For measurement of NO using diaminofluorescein-FM diacetate (DAF-FM DA), cells were incubated with 10 μM DAF FM-DA and 10 μM BAPTA-AM in 450 μL KRB Buffer (89 mM NaCl, 4.7 mM KCl, 25 mM NaHCO_3_, 1.2 mM MgSO_4_-7H_2_O, 1.2 mM KH_2_PO_4_, 2.5 mM CaCl_2_-2H_2_O, 11.1 mM D-Glucose, 20 mM Hepes-Na, pH7.4) for 1 hr, then incubated with CCCP (10 μM) for 1 hr, and finally cells were harvested using cold 1 mL PBS containing 5 mM EDTA and NO was measured by flow cytometry^27^.

### Phosphorylation assay and mitochondrial fractionation

Phos-tag Western blot assay was performed as previously described^6^. Briefly, phospho-parkin was separated on Phos-tag or normal gels and then immunoblotted using anti-parkin antibody.

### [Ca2+]_i_ response analysis

Cells were seeded at a density of 2 × 10^5^ cells/well on collagen-coated 96-well plates, incubated at 37°C for 21 hr, and then incubated in Hanks' Balanced Salt Solution (HBSS, pH7.4) containing Calcium Assay Kit Component A (Molecular Devices, Sunnyvale, CA, USA) for 1 hr at room temperature. Compounds used in the fluorometric imaging plate reader (FDSS7000, Hamamatsu Photonics, Shizuoka, Japan) assay were dissolved in HBSS (1% DMSO) and they were prepared in another set of 96-well plates. These plates were set on the FDSS7000, and mobilization of [Ca2+]_i_ evoked by drugs was monitored^28^.

### Statistical analyses

Values are presented as the mean ± standard error (SEM). Differences between groups were examined for statistical significance using Student's *t*-test. *P* values < 0.05 were considered statistically significant.

## Author Contributions

K.O. designed the project. K.O., A.T.K. and H.K. performed the experiments and interpreted the data. Y.N. and T.T. performed the experiments using zebrafish and interpreted the data. T.S. and R.T. performed the immunostaining experiments to evaluate the mitochondrial degradation and interpreted the data. K.O. wrote the manuscript and A.T.K., Y.N., T.S., H.K., H.T., Y.T., J.Z., Y.K., T.T., R.T. and M.Y. edited the manuscript.

## Supplementary Material

Supplementary Information Supplemental Figures and Discussion
