# Supplementary material for: S-nitrosylation regulates mitochondrial quality control via activation of parkin
Source: Sci Rep. 2013 Jul 16;3:2202. doi: 10.1038/srep02202 (PMC3712319; doi:10.1038/srep02202)
Supplement: Supplementary Information — Supplemental Figures and Discussion [file srep02202-s1.pdf]

Supplemental Information

S-nitrosylation regulates mitochondrial quality control via activation of parkin.

Kentaro Ozawa[1*], Akira T. Komatsubara[1, 2], Yuhei Nishimura[3, 4, 5, 6], Tomoyo Sawada[7], Hiroto Kawafune[1, 2], Hiroki Tsumoto[8], Yuichi Tsuji[1], Jing Zhao[1], Yoji Kyotani[1], Toshio Tanaka[3, 4, 5, 6], Ryosuke Takahashi[7], Masanori Yoshizumi[1]

1. Department of Pharmacology, Nara Medical University School of Medicine, Japan.

2. Department of Genomic Drug Discovery Science, Kyoto University Graduate School of Pharmaceutical Sciences, Sakyo-ku, Kyoto, Japan

3. Department of Molecular and Cellular Pharmacology, Pharmacogenomics and Pharmacoinformatics, Mie University Graduate School of Medicine, Tsu, Mie, Japan

4. Mie University Medical Zebrafish Research Center, Tsu, Mie, Japan

5. Department of Bioinformatics, Mie University Life Science Research Center, Tsu, Mie, Japan

6. Department of Omics Medicine, Mie University Industrial Technology Innovation Institute, Tsu, Mie, Japan

7. Department of Neurology, Kyoto University Graduate School of Medicine, Kyoto, Japan

8. Research Team for Mechanism of Aging, Tokyo Metropolitan Institute of Gerontology, Tokyo 173-0015, Japan

Corresponding Author: Kentaro Ozawa, M.D., Ph.D.

Department of Pharmacology, Nara Medical University School of Medicine

840 Shijo-cho, Kashihara, Nara, 634-0813, Japan

TEL: +81-744-29-8831

FAX: +81-744-29-0510

E-mail: ko12@naramed-u.ac.jp

Supplemental Figure legends.

Supplemental Figure S1. Rotenone and CCCP regulate autoubiquitination of parkin. SH-SY5Y cells were transfected with the FLAG-tagged wild-type parkin expression plasmid and then treated with 1 μM rotenone (a) or 10 μM CCCP (b) in the presence of MG132 (10 μM) for the indicated times. Lysates were used in immunoprecipitation with anti-FLAG antibody, and immunoprecipitates were immunoblotted with anti-ubiquitin (upper panel) or anti-FLAG (middle panel) antibody and lysates were immunoblotted with anti-ubiquitin as control (lower panel). The quantity of ubiquitinated parkin, as measured by scanning densitometry, is expressed as a percentage of control, normalized with respect to total parkin and total ubiquitinated proteins. Data shown are mean ± SE (n = 3); **p < 0.01 versus control.

Supplemental FIgure S1

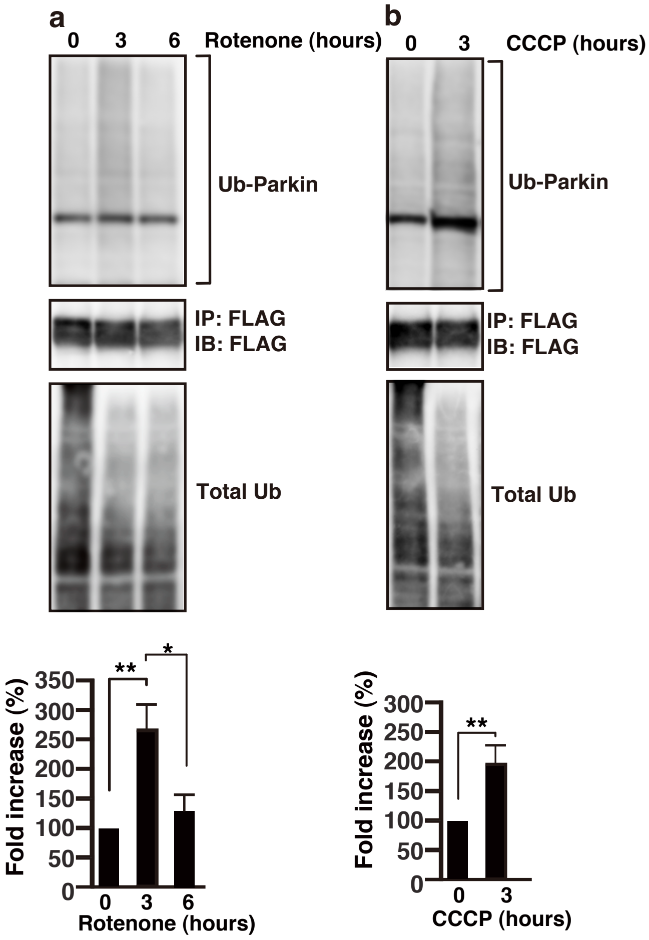

Supplemental Figure S2. HeLa cells expresses negligible amount of parkin compared to that in SH-SY5Y cells. HeLa cells and SH-SY5Y cells were transfected with the control plasmid or FLAG-tagged wild-type parkin expression plasmid, and cell lysates were immunoblotted with anti-FLAG, anti-parkin, anti-nNOS and anti-beta-actin antibodies. We repeated the experiment three times and the representative results are shown.

Supplemental FIgure S2

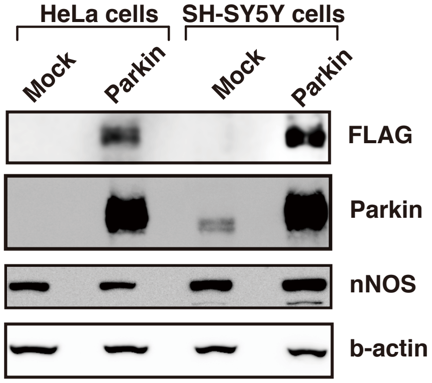

Supplemental Figure S3. Cys323 is the S-nitrosylated cysteine in parkin. (a) SH-SY5Y cells were transfected with the plasmid harboring FLAG-tagged wild-type (full-length) parkin (wt: amino acids 1–465 of the human parkin open reading frame) or truncated parkin (amino acids 191–465, 291–465, 1-300 or 1–360), and cell lysates were analysed by SNO-RAC as described before, followed by immunoblot analysis with anti-FLAG antibody (upper panels). Lower panel shows a diagram depicting the parkin constructs used in the experiment. RING, Ring-finger motif; IBR, in-between-Ring fingers domain. This experiment was repeated three times and the representative results are shown. (b) SH-SY5Y cells were transfected with the plasmid expressing FLAG-tagged wild-type (full-length) parkin or truncated parkin (amino acids 191–465, 242–465 or 269–465), and cell lysates were analysed by SNO-RAC as before, followed by immunoblot analysis with anti-FLAG antibody (upper panels). Lower panel shows a diagram of parkin constructs used in this experiment. We performed the experiment three times and the representative results are shown. (c) SH-SY5Y cells were transfected with the plasmid expressing the FLAG-tagged wild-type parkin or a cysteine-to alanine mutant of parkin (C268A, C289A, C323A, C332A or C337A), and cell lysates were analysed by SNO-RAC as before, followed by immunoblot analysis with anti-FLAG antibody. We performed the experiment three times and the representative results are shown. (d) SH-SY5Y cells were transfected with the plasmid expressing the FLAG-tagged wild-type, a disease-related mutant of parkin (K161N, K211N or T415N) or C323S mutant of parkin, and cell lysates were analysed by SNO-RAC as before, followed by immunoblot analysis with anti-FLAG antibody. We performed the experiment three times and the representative results are shown. (e) SH-SY5Y cells, transfected with the plasmid expressing the FLAG-tagged wild-type, C323A or C323S mutant of parkin, were incubated with rotenone (1 µM) for 6 hr in the presence of MG132 (10 µM), and then cell lysates were analysed by SNO-RAC as before (upper panels). The quantity of S-nitrosylated parkin, as measured by scanning densitometry, is expressed as a percentage of rotenone-untreated wild-type, normalized with respect to total parkin. Data shown are mean ± SE (n = 3); **$p < 0.01$ (lower panels).

Supplemental FIgure S3

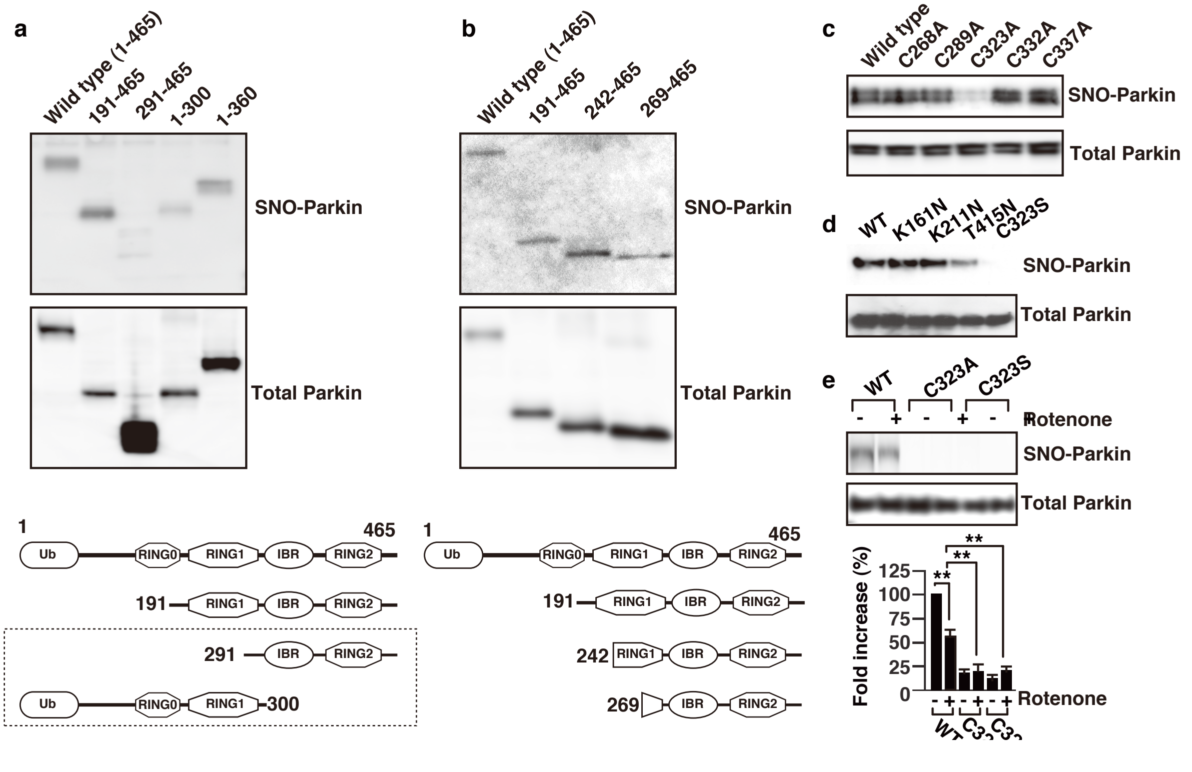

Supplemental Figure S4. Effect of S-nitrosylation on the activity of parkin. HeLa cells were transfected with the FLAG-tagged wild-type or C323S mutant expressing plasmid and then treated with GSNO (50 μM) for 3 hr in the presence of MG132 (10 μM). Lysates were used in immunoprecipitation with anti-FLAG antibody, and immunoprecipitates were immunoblotted with anti-ubiquitin (upper panel) or anti-FLAG (middle panel) antibody and lysates were immunoblotted with anti-ubiquitin as control (lower panel). We performed the experiment two times and the representative results are shown.

Supplemental FIgure S4

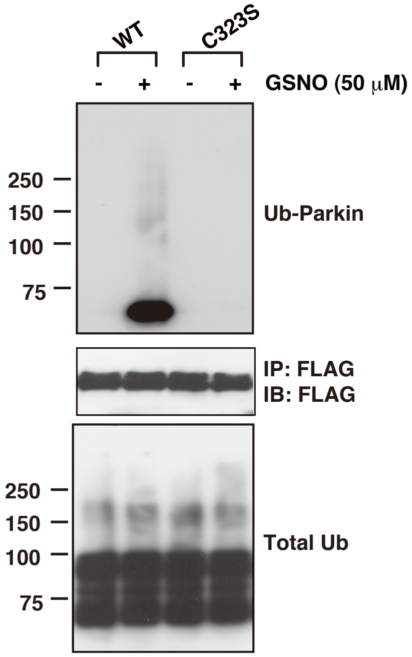

Supplemental Figure S5. The effect of S-nitrosylation on mitochondrial degradation. (a) HeLa cells transfected with the Venus-tagged wild-type or C323S mutant expressing plasmid were incubated with GSNO (50 μM) and/or CCCP (10 μM) for different time periods as indicated, and then the cells were immunostained with anti-Tom20 and anti-HSP60 antibodies. The dotted line surrounded a parkin-expressing cell. Scale bars = 10 μm. (b) After preincubation with L-NMMA (500 μM) and L-Arginine (1 mM), HeLa cells transfected with the plasmid expressing the Venus-tagged wild-type or C323S mutant were incubated with CCCP (10 μM) in the presence of L-NMMA (500 μM) and L-Arginine (1 mM) for 3 hr, and then the cells were immunostained with anti-Tom20 antibody. The dotted line surrounded the parkin-expressing cells. Scale bars = 10 μm.

Supplemental FIgure S5

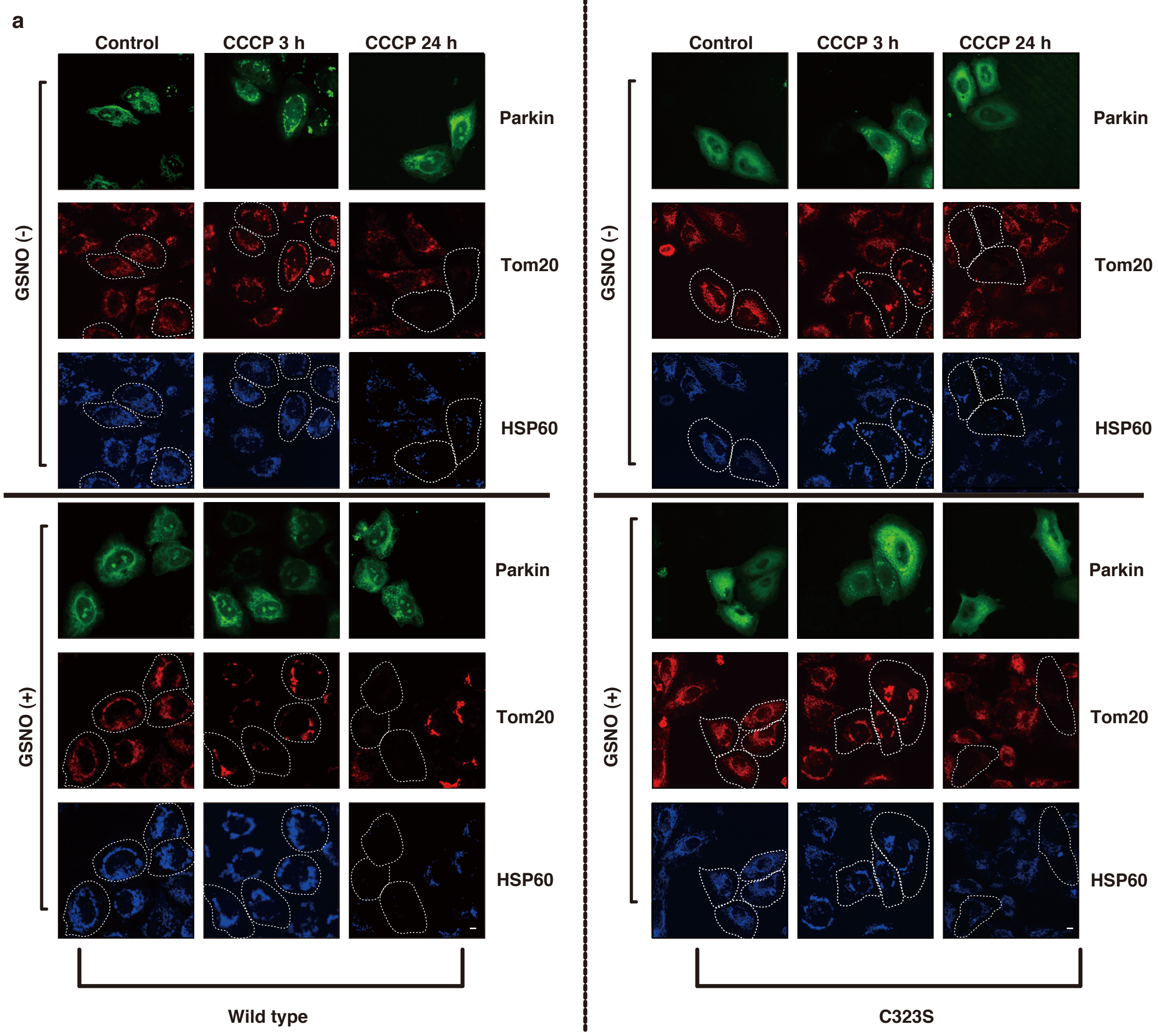

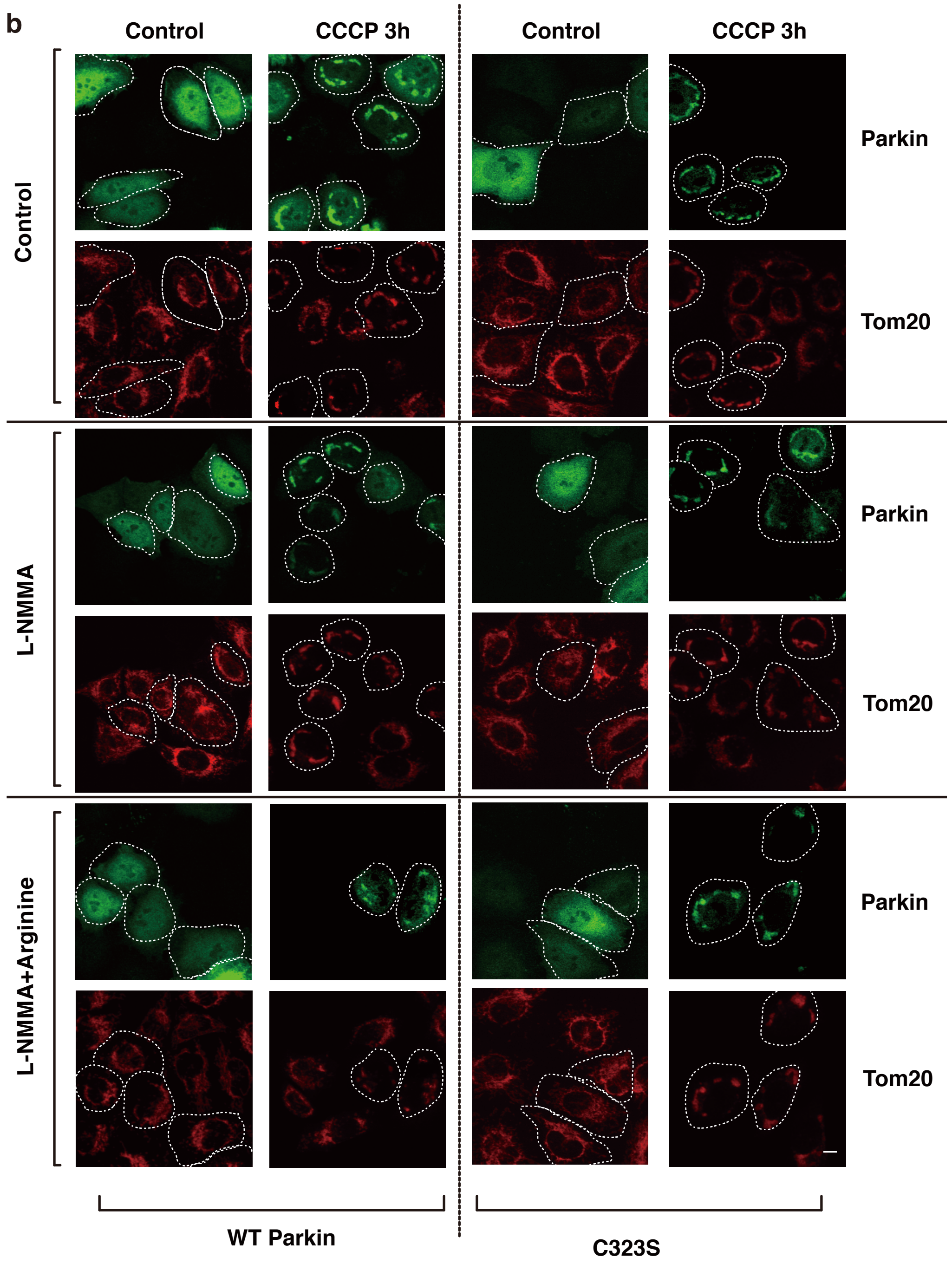
b
Control
CCCP 3h
Control
CCCP 3h
Control
L-NMMA
L-NMMA+Arginine
Parkin
Tom20
Parkin
Tom20
Parkin
Tom20
WT Parkin
C323S

Supplemental Figure S6. S-nitrosylation of parkin has no effect on parkin phosphorylation or recruitment of parkin to mitochondria. (a) HeLa cells were transfected with the wild-type or C323S parkin expressing plasmid and were treated with or without 10 μM CCCP for 1 hr. Cell lysates were subsequently separated on a Phos-tag gel (upper panel) or a normal gel (lower panel), followed by immunoblotting with anti-parkin antibody. We performed the experiment three times and the representative results are shown. (b) HeLa cells transfected with the plasmid expressing the Venus-tagged wild-type or C323S parkin were treated with or without 10 μM CCCP for 1 hr, and then the cells were immunostained with anti-Tom20 antibody (upper panel). Scale bars = 10 μm. Obtained images were analysed using ImageJ (NIH) and recruitment of parkin to the mitochondria was calculated as a percentage of parkin signals overlapping with Tom20 to total parkin signals (lower panels). Data shown are mean ± SE (n = 5); $*p < 0.05$ and $**p < 0.01$. N.S. means no significant difference.

Supplemental FIgure S6

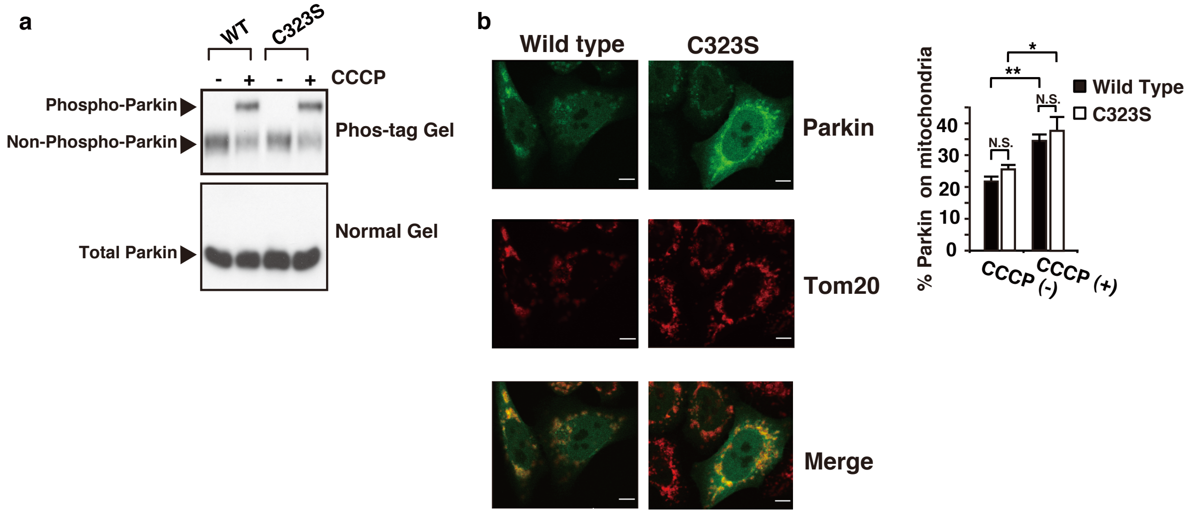

Supplemental Figure S7. CCCP and rotenone increase cytosolic calcium concentration and NO production. (a) and (b) Cytosolic calcium concentrations in SH-SY5Y cells treated with rotenone (a) or CCCP (b) were measured. (c) After preincubation with 10 μM BAPTA-AM for 1 hr, SH-SY5Y cells were incubated with CCCP (10 μM) for 1 hr, and then the generated NO was measured by flow cytometry using DAF (10 μM) as described in Materials and Methods. Levels of NO were calculated as relative fold-changes in fluorescence compared to the CCCP-untreated control. Data shown are mean ± SE (n = 5); *p < 0.05 versus wild type. N.S. means no significant difference.

Supplemental FIgure S7

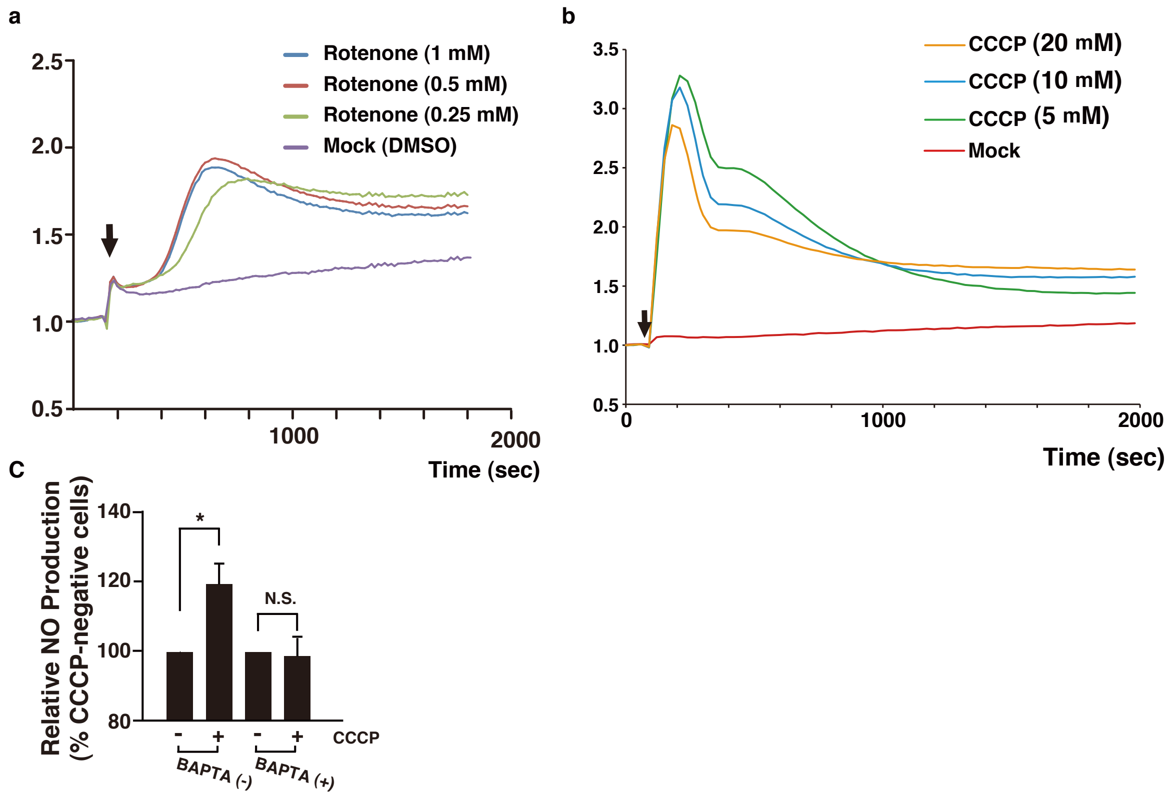

Supplemental Figure S8. Nitrotyrosines are condensed around the mitochondria in C323S-expressing cells. HeLa cells overexpressing the wild-type and C323S mutant of parkin were incubated with CCCP for 3 hr and then immunostained with anti-Tom20 and anti-Nitrotyrosine antibodies. White arrows indicate condensed nitrotyrosine. Scale bars = 10 μm.

Supplemental FIgure S8

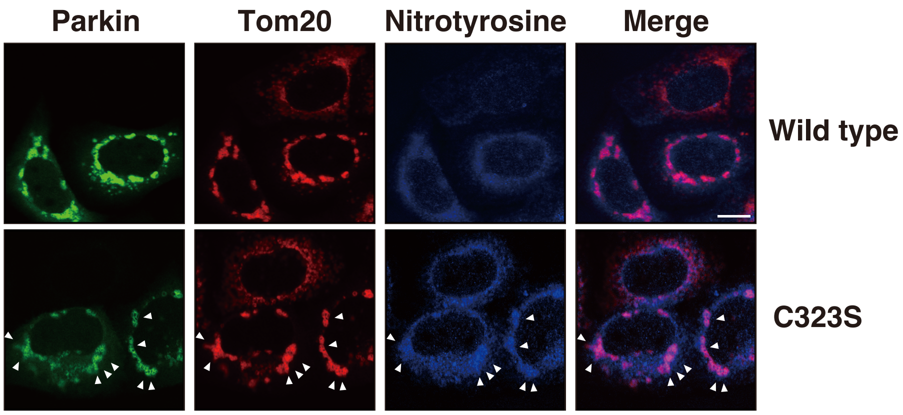

Supplemental Figure S9. Schematic summary of the regulation of mitochondrial degradation by S-nitrosylation/denitrosylation and tyrosine-nitration of parkin.

(a) Mitochondrial depolarization upregulates cytosolic calcium concentration and subsequent activation of NOS, leading to S-nitrosylation of parkin. S-nitrosylation of parkin increases its E3 ligase activity, independently of the PINK1-dependent phosphorylation and recruitment of parkin to mitochondria. (b) Sustained mitochondrial depolarization produces superoxide, which reacts with NO to produce peroxynitrite, which in turn decreases S-nitrosylation of parkin (denitrosylation) and increases tyrosine nitration of parkin, leading to its inactivation.

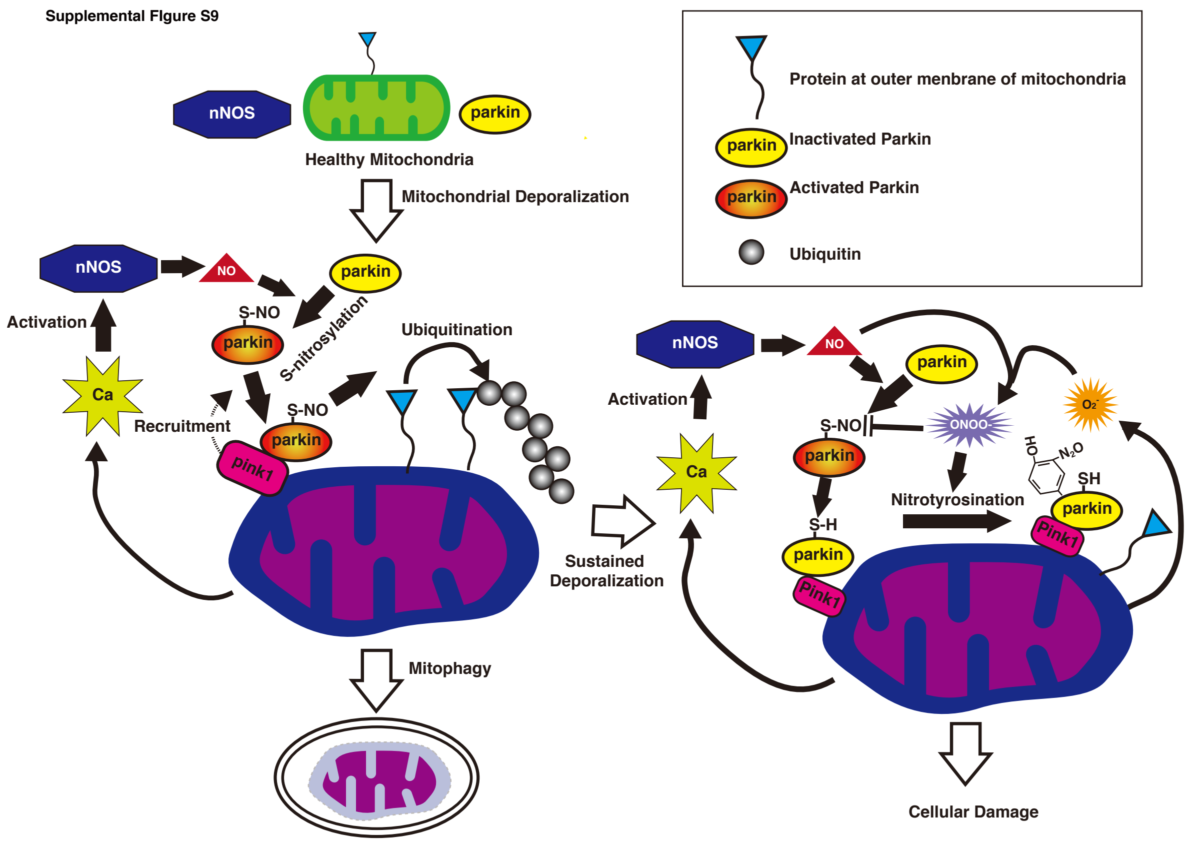

Supplemental Figure S10. Full scans of the blots shown in Figs. 1a, 1b, 1d, 2b, 2c, 2d, 2e, 2f, 3b, 4a, 4b, 5a, 6a, 6b, 6d and 6e.

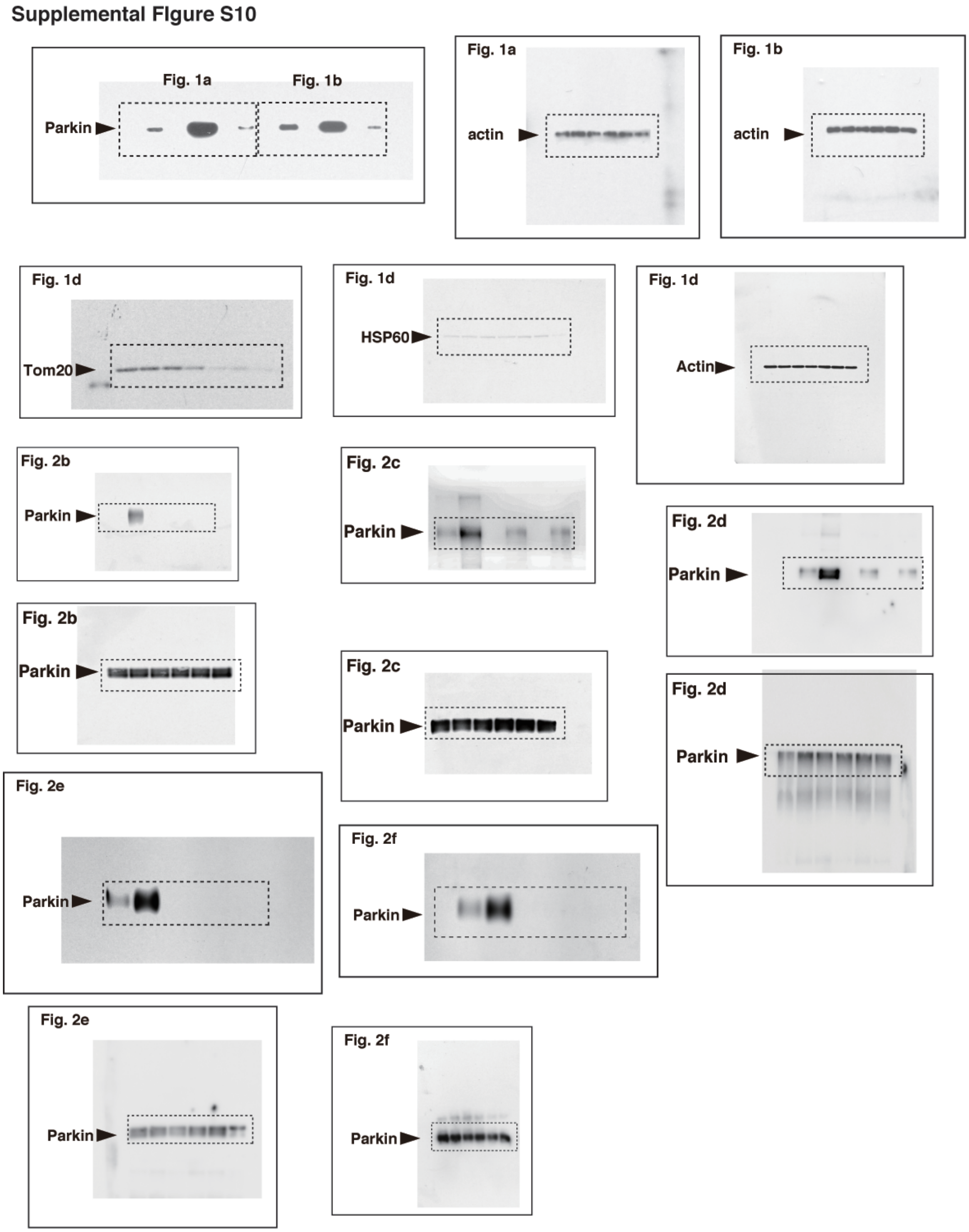

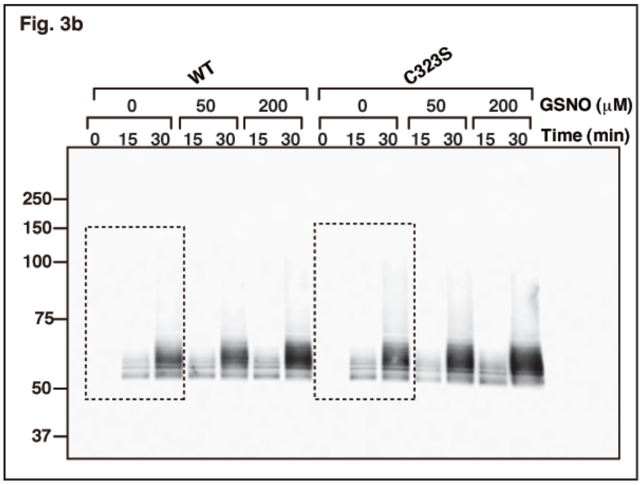

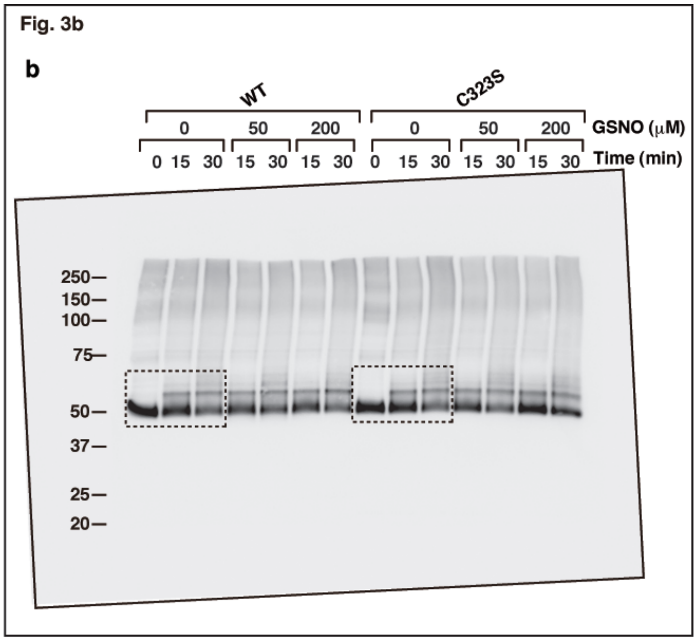

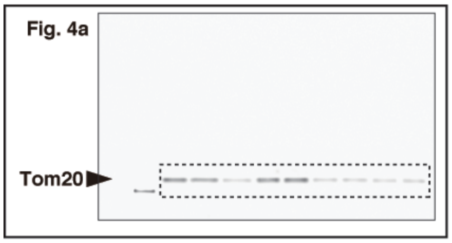

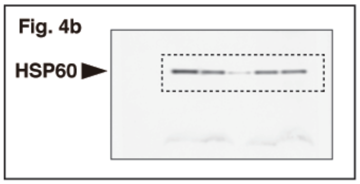

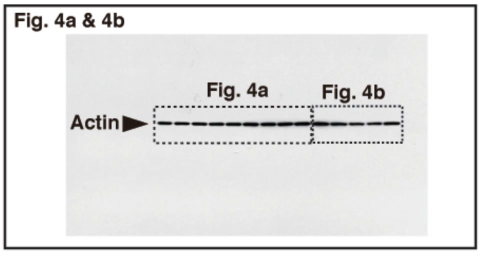

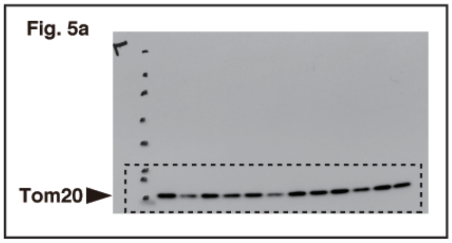

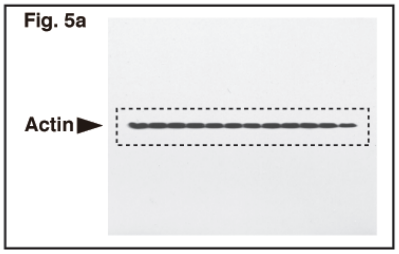

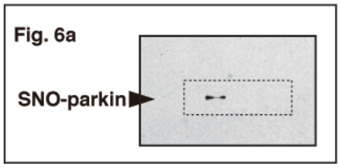

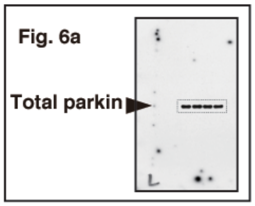

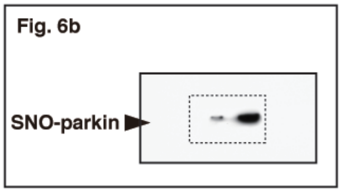

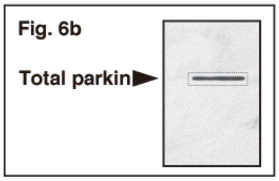

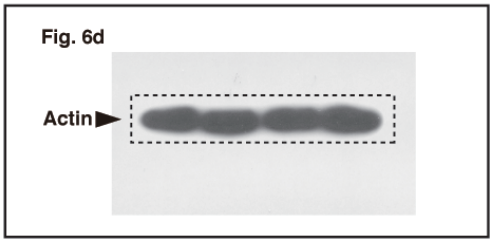

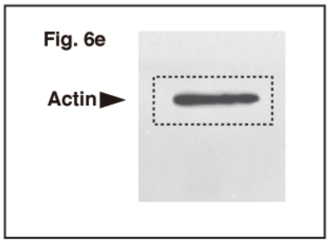

Supplemental FIgure S10 contiued.

Supplemental Discussion

S-nitrosylation of parkin was reported earlier by two groups, Dawson's group [s1] and Lipton's group [s2]; both group clearly showed that S-nitrosylated parkin is present at detectable levels in human brains of patients with PD and rodent model of PD created using MPTP. However, there are some discrepancies between the results observed by these two groups. Here, we have shown that Cys323 is the cysteine that gets S-nitrosylated in parkin and S-nitrosylation of parkin increased its activity. Our observations also differed from those of the other two groups. These observed discrepancies in results might be due to several technical differences.

The former two groups claimed multiple cysteines were S-nitrosylated, whereas we showed that Cys323 is exclusively S-nitrosylated in parkin. This discrepancy could be due to differently prepared samples used for identifying the S-nitrosylated residue. Dawson's group used cell lysates incubated with the NO-donor GSNO[s1] and Lipton's group used recombinant parkin exposed to S-nitrocysteine[s2] to find that multiple cysteines were S-nitrosylated in parkin. Under both of these experimental conditions, the S-nitrosylated cysteine residue(s) might be different from the one(s) that is(are) S-nitrosylated under the physiological or pathophysiological condition. We, on the other hand, used site-directed mutagenesis and showed that the Cys323 residue in parkin was predominantly S-nitrosylated under the basal condition in neuroblastoma cells and that S-nitrosylation of parkin at Cys323 regulated its E3 ligase activity. Furthermore, we showed that under pathological conditions other cysteine(s) could be S-nitrosylated, but to a much lesser extent than Cys323.

Dawson's group observed that S-nitrosylation reduced the E3 ligase activity of parkin[s1], whereas both Lipton's group[s2] and we (this study) observed that S-nitrosylation increased the E3 ligase activity of parkin following short-time treatment with drugs. There might be two reasons for the observed discrepancy in results. First, regulation of ligase activity by S-nitrosylation/denitrosylation is time-dependent. As Lipton's group pointed out correctly[s3], Dawson's group evaluated the level of ubiquitination 6 hr after the treatment of the drug. We showed that treatment with GSNO for 3 hr increased the activity of parkin, whereas longer treatment decreased its activity to the basal level. In addition, level of S-nitrosylated parkin after 6 hr treatment with rotenone or CCCP was less than the basal level (denitrosylation), suggesting that denitrosylation, and not S-nitrosylation, might be the cause behind the reduced E3 ligase activity of parkin.

NO regulates diverse pathways through multiple pathways and mechanisms including S-nitrosylation and production of peroxynitrite. Dawson's group used NO-donor and NOS inhibitor to investigate the regulation of parkin's activity by S-nitrosylation[s1]. It is, however, difficult to distinguish between S-nitrosylation from other NO-mediated mechanisms without definitive identification of the S-nitrosylated cysteine residue. To clarify the role of S-nitrosylation on parkin's function, we generated mutants in which the Cys323 was changed to an alanine or a serine residue, and compared the activities of the wild-type and mutant parkins. Our results showed that NO could not activate the E3 ligase activities of C323A and C323S mutants, which suggested that NO activate parkin via the Cys323 residue. FeTPPS, a peroxynitrite decomposition catalyst, on the other hand, enhanced the CCCP-induced degradation of mitochondria in cells overexpressing either the wild-type or the C323S parkin, thus suggesting that peroxynitrite, in addition to denitrosylation of parkin, also decreases parkin's

activity independent of Cys323.

In conclusion, results presented in this study suggested that S-nitrosylation of parkin at Cys323 activates parkin, and showed that introduction of mutations at this site also affects subsequent mitophagy processes. Concurrently, our data raise the possibility that there is an elaborate multi-step mechanism for the NO-mediated regulation of E3 ligase activity upon mitochondrial depolarization, the clarification of which requires further study.

Supplemental Reference

S1. Chung, K.K. ***et al.*** S-nitrosylation of parkin regulates ubiquitination and compromises parkin's protective function. *Science.* **304**, 1328-31 (2004).

S2. Yao, D. ***et al.*** Nitrosative stress linked to sporadic Parkinson's disease: S-nitrosylation of parkin regulates its E3 ubiquitin ligase activity. *Proc. Natl. Acad. Sci. U. S. A.* **101**, 10810-4 (2004).

S3. Lipton, S.A. ***et al.*** Comment on "S-nitrosylation of parkin regulates ubiquitination and compromises parkin's protective function". *Science.* **308**, 1870 (2005)
